# A Review of Artificial Intelligence in Breast Imaging

**DOI:** 10.3390/tomography10050055

**Published:** 2024-05-09

**Authors:** Dhurgham Al-Karawi, Shakir Al-Zaidi, Khaled Ahmad Helael, Naser Obeidat, Abdulmajeed Mounzer Mouhsen, Tarek Ajam, Bashar A. Alshalabi, Mohamed Salman, Mohammed H. Ahmed

**Affiliations:** 1 Medical Analytica Ltd., 26a Castle Park Industrial Park, Flint CH6 5XA, UK; shakir@russellipm.com; 2 Royal Medical Services, King Hussein Medical Hospital, King Abdullah II Ben Al-Hussein Street, Amman 11855, Jordan; khalid@medicalanalytica.co.uk; 3 Department of Diagnostic Radiology and Nuclear Medicine, Faculty of Medicine, Jordan University of Science and Technology, Irbid 22110, Jordan; nmobeidat8@just.edu.jo (N.O.); abma10695@gmail.com (A.M.M.); tkajam20@med.just.edu.jo (T.A.); alshalabi666@gmail.com (B.A.A.); mohammed25158@gmail.com (M.S.); 4 School of Computing, Coventry University, 3 Gulson Road, Coventry CV1 5FB, UK; ae2333@coventry.ac.uk

**Keywords:** artificial intelligence network, deep learning, machine learning, breast cancer, ultrasound image, mammography image

## Abstract

With the increasing dominance of artificial intelligence (AI) techniques, the important prospects for their application have extended to various medical fields, including domains such as in vitro diagnosis, intelligent rehabilitation, medical imaging, and prognosis. Breast cancer is a common malignancy that critically affects women’s physical and mental health. Early breast cancer screening—through mammography, ultrasound, or magnetic resonance imaging (MRI)—can substantially improve the prognosis for breast cancer patients. AI applications have shown excellent performance in various image recognition tasks, and their use in breast cancer screening has been explored in numerous studies. This paper introduces relevant AI techniques and their applications in the field of medical imaging of the breast (mammography and ultrasound), specifically in terms of identifying, segmenting, and classifying lesions; assessing breast cancer risk; and improving image quality. Focusing on medical imaging for breast cancer, this paper also reviews related challenges and prospects for AI.

## 1. Introduction

Breast cancer is one of the most common cancers identified in women across the globe, and it is now the leading cause of death among women [1,2,3]. In addition to being the most commonly diagnosed cancer in 154 countries (out of 185 countries), breast cancer is the main cause of cancer-related death in more than 100 countries [4]. Approximately 600 men and 40,000 women die of breast cancer annually according to the findings of the American Cancer Society [2]. Therefore, early screening and treatment of breast cancer is a global health concern. Accurate diagnosis of breast cancer, especially early detection and treatment, can critically affect its prognosis. The clinical cure rate of breast cancer in the early stage is highly optimistic and exceeds 90%. Meanwhile, the clinical cure rate in the middle stage ranges from 50% to 70%, and treatment is typically not effective for those in the late stage. Mammography, ultrasound, and MRI are crucial screening and supplementary diagnostic tools that serve as significant methods for the detection and staging of breast cancer, evaluation of treatment efficacy, and follow-up examination [5]. Breast cancer can be classified as benign, normal, in situ carcinoma, or invasive carcinoma [3]. A benign tumor will cause a slight alteration to the breast anatomy; however, there is no harmful substance, so it cannot be referred to as a dangerous tumor [6]. On the other hand, in situ carcinomas affect only the mammary duct lobules and do not spread to other tissues [7]. As long as it is detected at an early stage, this type of cancer is not very dangerous and can be treated. Invasive carcinoma is the most severe form of breast cancer, as it can spread to every organ in the body [8].

AI refers to the capability of a computer system to accurately interpret and learn from external data and to flexibly adapt obtained knowledge to execute specific tasks. The remarkable advancements in computational functions related to the rise of big data over the past five decades have propelled the application of AI into new domains [9]. For example, AI is available for face and voice recognition, among other new technologies. The use of AI methods in medical imaging has gained increasing research interest. Significant progress has been made in applying AI algorithms, particularly deep learning (DL) algorithms, to image recognition tasks. The availability of various methods in the field of medical image analysis, ranging from convolutional neural networks (CNNs) to variational autoencoders, has contributed to rapid developments in medical imaging [10]. Furthermore, various types of cancers, such as ovarian cancer, can be diagnosed using AI and machine learning (ML) tools [11,12,13,14].

Radiologists read and analyze breast images and use them for diagnosis. However, their substantial workload and long hours can cause fatigue, leading to misjudgment and misdiagnoses or missed diagnoses. Using AI, or, in this case, computer-aided diagnosis (CAD), for this purpose can alleviate potential human errors. In a CAD system, a suitable algorithm completes the processing and analysis of images [15,16]. Recent breakthroughs with DL in AI, especially CNNs, have significantly advanced the field of medical imaging [17,18]. AI refers to the capability of applications or machines to replicate humans (or human brain functions) in order to learn and solve problems [19]. The concept of AI was introduced in 1956 by John McCarthy. AI technologies have made remarkable progress since then, especially over the past decade. As AI has become a key part of computer science, there have been continuous efforts to create new kinds of intelligent machines that can imitate the human brain, extending to diverse applications such as image recognition, data mining, expert systems, natural language processing, language recognition, pattern recognition, and robotics [20]. In the medical field, AI can be used for early disease prediction, disease screening, clinical decision support, health management, hospital management, medical imaging, and medical record or literature analysis. Apart from assisting doctors in making accurate diagnoses, AI can be used to analyze medical images and information for disease screening and prediction. Focusing on the application of AI in breast imaging, Al-antari et al. reported high accuracy for a complete integrated CAD system (>92%) in the detection, segmentation, and classification of masses seen on mammograms [21]. Based on 2654 exams and readings by 101 radiologists, Rodriguez-Ruiz et al. reported a significant reduction in radiologists’ workload (of 17%) when using a trained AI system with automatic preselection (with an AI score of 2 as the threshold when scoring the possibility of cancer from 1 to 10) [22].

**Figure 1:**
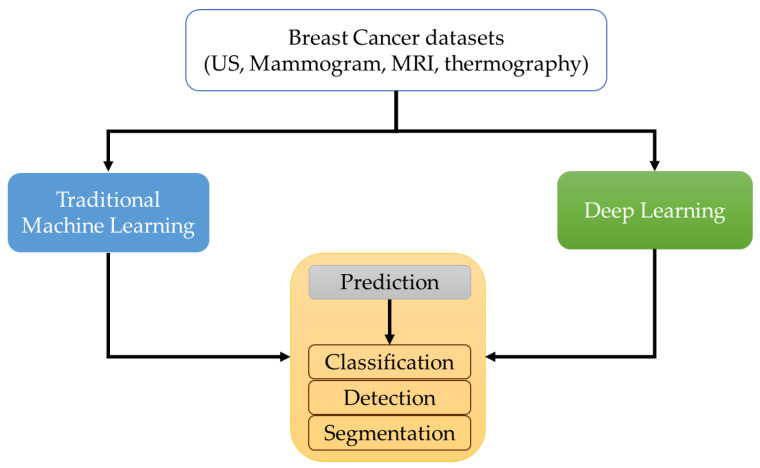
Overview of ML for breast cancer.

One of the most notable ways AI is used is in the implementation of ML, which consists of unsupervised and supervised ML techniques. Unsupervised ML does not require any information to be provided or determined by a set of historical imaging data, similar to the data under study, in order to categorize radiomic features, as shown in Figure 1. Supervised ML involves training using available data labeled with corresponding correct outputs. 

The selected method must reach a balance between its ability to fit the training set with its power to generalize in the case of new data; until then, all parameters in the algorithm are subjected to tuning. For instance, sparsity-enhancing regularization networks can concurrently make predictions and recognize extracted features that significantly affect predictions [22]. ML includes computational algorithms, such as artificial neural networks (ANNs), decision trees, *k*-means, linear regression, principal component analysis (PCA), random forests, and support vector machines (SVMs), which utilize the image features extracted by radiomics as input to produce output predictions concerning disease outcomes. As AI systems are based on neural networks, DL approaches involve developing models that can imitate the human brain, which is the most recent technology used for image classification. A simulation model known as a perceptron was the first model to be used to simulate neural networks and the human brain. A neural network has a variable number of layers. The input layer processes the multi-dimensional data used as input. The hidden layer consists of several layers. In a convolutional layer, a feature map is created and passed through a non-linear activation function before it reaches the pooling layer for down sampling. The output can then be transferred to a fully connected layer for classification. Finally, the output layer yields the results of the analysis. For more complex problem solving, layers with perceptrons with all nodes fully connected can be created and arranged to form a multi-layer perceptron [23]. In addition, CNNs can be either supervised or unsupervised learning models. Supervised learning is a model-training procedure that requires the observed training data and the associated ground truth labels for the data (also described as “objects”). In unsupervised learning, there are no diagnoses or normal/abnormal labels for the training data. Supervised learning is typically considered for image classification tasks [24]. This paper discusses AI (DL and ML) and its applications in medical imaging of the breast (mammography and ultrasound), as well as the challenges and prospects related to applying AI techniques to medical imaging.

## 2. Public Breast Cancer Datasets

This section presents some of the most common public datasets in both mammogram and ultrasound images. Researchers frequently employ machine learning techniques to analyze and derive insights from these datasets, facilitating advancements in the field of medical imaging. These datasets collectively contribute to the advancement of machine learning techniques in mammography, fostering innovation in breast cancer detection and diagnosis.

### 2.1. Public Mammography Datasets

In the realm of mammography research, several publicly available datasets serve as crucial resources for advancing machine learning techniques. These datasets offer annotated information and diverse images, enabling researchers to develop and evaluate algorithms effectively. Here, we highlight some of the prominent public mammography datasets:

MIAS (Mammographic Image Analysis Society) [25]: This dataset comprises over 300 screening mammograms annotated with information about background tissue type, abnormalities present in the breast, and their severity. Additionally, lesions in mammograms are marked with X and Y coordinates, along with labels for various abnormalities.

DDSM (Digital Database of Screening Mammography) [26]: With over 2600 scanned film mammography studies, the DDSM provides a comprehensive archive. A subset of the DDSM, known as the CBIS-DDSM, offers well-annotated images with detailed pathological information such as breast mass type, tumor grade, and stage.

INBreast Database [27]: Despite recent discontinuation of support by the Universidade do Porto, INBreast remains a valuable resource, with over 410 screening mammographs. It includes information on abnormality types and mass contour data.

BCDR (Breast Cancer Digital Repository) [28]: This dataset focuses on full-field digital mammograms (FFDMs) and encourages contributions from researchers [29]. It provides images in both craniocaudal and medio-oblique views, accessible after registration.

BancoWeb LAPIMO [30]: Comprising over 1400 mammograms, this dataset offers images in TIFF format collected from 320 subjects. It includes a variety of lesions classified as benign, malignant, or healthy.

VICTRE Trial dataset [31]: Unlike the others, this dataset is entirely synthetic, simulating 2986 subjects with representative breast sizes and densities. It uses in-silico versions of digital breast tomosynthesis (DBT) and provides open-source software tools for analysis and lesion insertion [32].

OPTIMAM dataset [31]: Available upon request from the University of Surrey, OPTIMAM offers relational data storage. It contains annotated 3D DBT imaging and provides an open-source Python package for easy integration into research systems, facilitating seamless processing.

### 2.2. Public Ultrasound Datasets

These datasets stand as pivotal resources frequently utilized by researchers in machine learning. Each dataset offers a distinct collection of breast ultrasound images, catering to various research needs.

BUS [33]: Sourced from the UDIAT Diagnostic Centre of the Parc Tauli Corporation in Sabadell, Spain, BUS comprises 163 breast ultrasound images. Among these, 109 depict benign conditions, while 54 exhibit malignant characteristics.

BUSI [34]: Gathered from the Baheya Hospital for Early Detection and Treatment of Women’s Cancer in Cairo, Egypt, BUSI features a more extensive collection. This dataset encompasses ultrasound images obtained from 600 female patients aged between 25 and 75 years. It comprises 437 benign images, 210 malignant images, and 133 images depicting normal breast conditions, totaling 730 ultrasound images.

BUSIS [35]: Originating from the Second Affiliated Hospital of Harbin Medical University, the Affiliated Hospital of Qingdao University, and the Second Hospital of Hebei Medical University, BUSIS consists of 562 images depicting female subjects aged 26 to 78 years. Notably, these datasets may contain multiple images representing the same patient.

**Figure 2:**
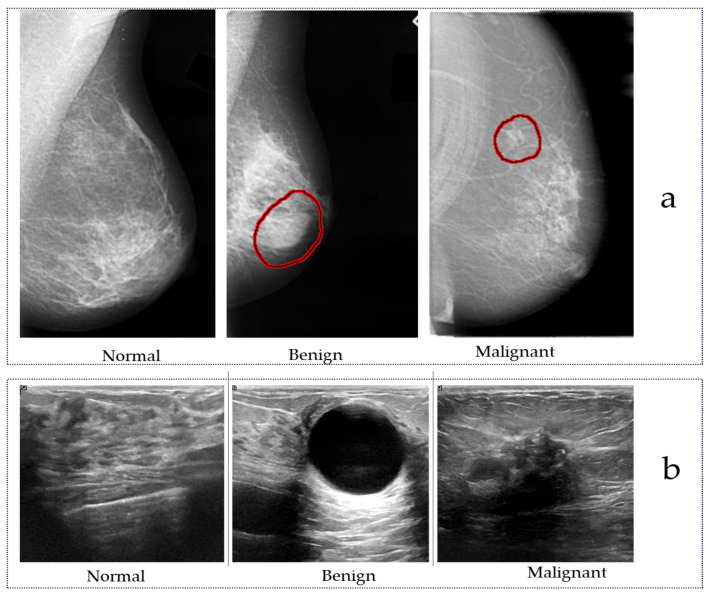
(**a**) A sample from the DDSM dataset [26], and (**b**) a sample of breast ultrasound images from the dataset [34].

Regarding image labels, both BUS and BUSI provide lesion shape labels along with classifications distinguishing between benign and malignant conditions. In contrast, BUSIS solely offers lesion shape labels without further classification details. Figure 2 illustrates mammogram and ultrasound images from public datasets.

## 3. Applying AI to Mammography

Breast cancer screening methods often involve the use of mammography [36]. This non-invasive screening method is easy to operate and yields high-resolution images with good repeatability. It is relatively pain-free and does not discriminate by age or body type. The retained images can also be compared (i.e., before vs. after). Breast masses that cannot be detected by touch, benign lesions, and malignant breast tumors can be accurately detected using mammograms. Mammography involves full-field digital mammography (DM) systems and yields for-processing (raw imaging data) and for-presentation (post-processed versions of raw data) image formats [37]. The use of AI for detecting, classifying, and segmenting breast masses, assessing breast cancer risk, and improving image quality are discussed in the following subsections (see Table 1).

### 3.1. Detection and Classification of Breast Masses

One of the most common symptoms of breast cancer is the presence of masses. Detecting and diagnosing such abnormalities can be challenging, especially in dense breasts, due to their different shapes, sizes, and margins. This is why breast mass detection is a pivotal step in CAD. Several studies have recommended crow search optimization based on an intuitive fuzzy clustering approach with neighborhood attraction (CrSA-IFCM-NA). Its effectiveness in separating masses in mammogram images and good outcomes on cluster validity indices, indicating precise region segmentation, has been proven [38]. Other studies recommend using a completely integrated CAD system comprising a regional DL approach (you only look once, YOLO), a new deep network model of a full-resolution convolutional network (FrCN), and a deep CNN for detection, segmentation, and classification of masses in mammograms, respectively. Using the INbreast dataset (2022), these studies reported remarkable detection accuracy of 98.96%, suggesting the potential for this system to assist radiologists in effectively and accurately diagnosing these masses [39,40].

Ertosun et al. [41] presented a CNN-based visual search approach designed to localize masses within mammograms. Their model comprises two key sub-modules: an anomaly detector followed by a mass localizer. Initially, the anomaly detector discerns whether a mammogram contains masses, subsequently channeling mass-containing images into the localizer for precise localization. These modules, leveraging hierarchical CNN layers, undergo training on a dataset exceeding 2500 images sourced from DDSM. Al-Masni et al. [42] opted for the you only look once (YOLO) deep network introduced by Redmon et al. [43] for simultaneous mass detection and classification. YOLO employs end-to-end learning, employing successive convolutional layers to segment the image into sub-regions, followed by placing bounding boxes around significant objects and assigning class labels. Following five-fold cross-validation, the authors reported impressive sensitivity (100%) and specificity (94%) scores on the INBreast dataset.

**Table 1:** An overview of key AI studies in mammography.

Ref.	Method	Application	Dataset Size	Accuracy
[37]	DoG and HoG	Microcalcification cluster detection	373 cases	-
[41]	CNN	Classification engine and a localization engine	2420 cases	85%
[42]	YoLo-based	Detection	600 cases	99.7%
[44]	Fast-RCNN	Detection	DDSM 2620 cases, SU-D 847 cases, INbreast 115 cases	-
[45]	Deep multi-instance networks	Classification	410 cases	90%
[46]	CBR	Classification	2620 cases	91.34%
[47]	CNN	Classification	INbreast 89 cases, MCA 49 case	90%
[48]	CNN features + MSVM	Classification	416 cases	90%
[49]	Deep fusion learning	Classification	208 cases	89.06%
[50]	Fuzzy contours	Segmentation	57 cases	88.08%
[51]	Mesh-free + SVM	Segmentation	322 cases	94.77%
[52]	Dense U-Net + AGs	Segmentation	D-A 186 cases, D-B 163 cases	78.38%
[53]	Mask-RCNNs + GCNNs	Segmentation	MIAS 58 cases, DDSM 200 cases	99.01%
[54]	CNN	Segmentation	885 cases	91%
[55]	Densely connected U-Net with attention gates (AGs)	Segmentation	400 cases	78.38%
[56]	Mask-RCNN with GCNN	Segmentation	MIAS 322 cases and INbreast 115 cases	99.1%
[57]	CLAHE and CNN	Image enhancement	DDSM 6000 cases, ZMDS 1739 cases	85.5%
[58]	FADHECAL and FCIS	Image enhancement	DDSM 2620 cases, MIAS 322 cases	-
[59]	LH and FEF	Image enhancement	97 cases	-

Ribli et al. [44] deployed a Faster R-CNN approach, achieving a commendable AUC of 0.95 on the INBreast dataset. Notably, they contribute to reproducibility by open-sourcing their implementation on GitHub, a rare practice in the field. To enhance model robustness, the authors train on DDSM data and evaluate on INBreast, demonstrating domain generalizability. Platania et al. [45] employed pretrained CNN weights to initialize a binary classifier’s weights in a semi-supervised manner. Their approach involves a two-module system, where a YOLO-inspired CNN detects regions of interest (ROIs). Subsequently, the initial detector’s weights are transferred to an FFDM classifier, which undergoes training on the entire mammogram image. Post-testing on DDSM, the authors report an AUC score of approximately 92.3% and an accuracy of 93.5%.

In another work [46], the authors proposed a novel framework for detecting breast cancer in mammogram images. The images are classified in an explainable manner using the proposed solution. In particular, a classification approach based on case-based reasoning (CBR) was used. The quality of the extracted features strongly influences the accuracy of CBR. Therefore, a pipeline was developed to improve the quality of extracted features and provide a final diagnosis. Mammogram images are segmented using a U-Net architecture, which is an efficient method for extracting regions of interest (RoIs). A mammogram segmented with DL provides accurate results, while a mammogram classified with CBR provides accurate and explainable results. This approach outperformed some famous ML and DL methods on the curated breast imaging subset of the digital database for screening mammography (CBIS-DDSM) (by 86.71% and 91.34%, respectively). A shallow–deep CNN was proposed by Gao et al. [47] to classify masses as benign or cancerous based on mammography images. Low-energy images are recombined using shallow CNNs, and unique features are extracted from these images using deep CNNs. Using their proposed technique, they achieved an accuracy of 90%.

A hybrid technique for breast cancer diagnosis was proposed and tested in [48]. Three DL CNN models were applied as feature extractors in this study: Inception-V3, ResNet50, and AlexNet. The term variance (TV) feature selection algorithm was used in this method in order to extract useful features from the CNN models. TV in statistics is a measure of the spread or dispersion of a set of values. It quantifies how far each data point in the set is from the mean (average) and provides insight into the variability or volatility of the data. After the TV-selected CNN features are combined, further selection is performed to determine which features are most useful, and those features are then passed to a multi-class support vector machine (MSVM). The Mammographic Image Analysis Society (MIAS) image database, specifically the mini-Mammographic Image Analysis Society (mini-MIAS) dataset, was used to test the effectiveness of the suggested method. Patches were assigned to the RoIs of the mammograms. After testing various TV feature subsets, the 600-feature subset with the best classification performance was identified. Compared with previous studies, this work achieved a higher classification accuracy (CA). Typical CA was 97.81% for 70% of data for training, 98% for 80% of data for training, and 99% for 90% of data for training. As a final step, ablation analysis was performed to emphasize the key parameters of the proposed network.

Yu, X et al. [49] investigated the discriminative patterns between normal and tumor categories based on deep fusion learning. The framework for mammographic image classification using deep fusion learning includes two steps. The proposed deep fusion models are first trained on RoI patches randomly chosen from all RoIs in the original dataset once they have been obtained. The authors developed a deep fusion model (Model 1) to classify the RoI patches in normal and tumor tissues. Another model (Model 2) integrates cross-channel deep features using one-to-one convolution to explore associations between channels of the same block. Patches that predict a majority vote make up one RoI and the final prediction. Model 1 achieved a recall rate of 0.913, a precision rate of 0.8077, and an overall accuracy rate of 0.8906, while Model 2 achieved an overall accuracy of 0.875, a recall rate of 0.9565, and a precision rate of 0.7586 for the tumor class data.

### 3.2. Segmentation of Breast Masses

The effectiveness of treatment directly depends on the correctness of the segmentation of masses. The use of fuzzy contours has been recommended for automatic segmentation of breast masses. One study recorded high average true positive (91.12%) and accuracy (88.08%) rates for RoIs extracted from the mini-MIAS dataset [50]. The low contrast of mammogram images, irregular shapes of masses, spiculated margins, and varying pixel intensities contribute to the complexity of global segmentation of masses in mammograms. In another study, the evolved level set function for segmentation of the breast and suspicious mass regions was explored using a mesh-free radial basis function collocation approach, and suspicious mass regions were classified into abnormal and normal using an SVM classifier, achieving high sensitivity (97.12%) and specificity (92.43%) on the DDSM [51]. Accurate segmentation of breast lesions ensures accurate disease classification and diagnosis [52]. Such automatic image segmentation algorithms demonstrate the promising potential for DL in precision medical systems.

A new segmentation method for tumor mammograms is presented in [53], which extracts both the spiculated regions and the mass core. There is a general linear pattern in the arrangement of pixels in spiculated regions, which is mirrored in mass core regions where the pixels are also linear. The proposed method extracts these regions based on the differences between adjacent pixels. In the mass core and spiculated regions, redundant pixels can be deleted using three thresholds; segmented tumors are then formed by merging these regions. This method presented a mean Dice coefficient of 0.9309 on the mini-MIAS dataset, and mean Jaccard coefficients of 0.9557 and 0.9132 on the DDSM. According to the results, when compared with other techniques, the proposed segmentation technique can accurately extract tumor segments.

Salama and Aly [54] collected images from the DDSM, the mini-MIAS dataset, and the CBIS-DDSM. They used a variety of models to segment and classify the images as benign or malignant, including DenseNet121, InceptionV3, VGG16, ResNet50, and MobileNetV2. When using InceptionV3 with data augmentation, the best accuracy was 88.87%.

Li et al. [55] proposed a fully automatic method combining densely connected U-Nets with attention gates (AGs) for breast mass segmentation on mammogram images. It consists of an encoder and a decoder: Convolutional networks encode information, and U-Nets integrated with AGs decode it. The DDSM, an authorized public screening database, was used to test the proposed method. The F1-score, meaning the intersection of union, sensitivity, specificity, and overall accuracy, was used to evaluate the effectiveness of the method. Compared with U-Net, attention U-Net, DenseNet, and state-of-the-art segmentation techniques, dense U-Net integrated with AGs outperformed the other methods and achieved better segmentation results, with an accuracy of 78.38% and an F1-score of 78.24%. Additionally, the network has a lower standard deviation, suggesting that it is more capable of generalization.

The results of the study reported in [56] demonstrated highly accurate breast cancer segmentation by combining mask regional CNNs (mask R-CNNs) with group CNNs (G-CNNs). These approaches maximize the share of weights and the expressive capacity of the model while maintaining rotational invariance. The INbreast and mini-MIAS datasets were used to test the model. Comparing the results with those of conventional architectures, the model achieved a 99.01% accuracy, a Dice coefficient of 86.63%, a Jaccard index of 87.76%, 99.24% sensitivity, and 98.55% specificity.

### 3.3. Image Quality Improvement

The accuracy of a diagnosis largely depends on the image quality. Good image quality means clear images, which significantly improve the diagnosis and accuracy rates of AI models when detecting and diagnosing microscopic lesions in mammograms. Various computer algorithms have been developed to enhance image quality. Higher image quality offers more information on the phase, directionality, and shift invariance of the data. In this regard, multi-scale shearlet transforms can produce multi-resolution results for the detection of cancer cells, particularly those with smaller contours. Shenbagavalli et al. reported that benign and malignant cases in the DDSM were classified with an accuracy of up to 93.45% using the shearlet transform image enhancement method, suggesting its effectiveness in enhancing mammogram image quality [15]. Teare et al. used a novel form of a false-color enhancement method to optimize the characteristics of mammograms through contrast-limited adaptive histogram equalization (CLAHE), and they utilized dual deep CNNs at different scales to classify the images and derivative patches, along with a random forest gating network. In this way, they achieved a sensitivity of 0.91 and a specificity of 0.80 [57]. Given the significance of image quality for accurate diagnosis, rigorous image quality evaluation and improvement are essential for subsequent analysis and diagnosis by ANN systems and radiologists.

To reduce the noise of mammogram images while preserving contrast and brightness, Suradi et al. [58] developed the fuzzy anisotropic diffusion histogram equalization contrast adaptive limited (FADHECAL) technique. In addition to the FADHECAL technique, a fuzzy clipped inference system (FCIS) is applied during the enhancement process, automatically selecting the clip limit from the available options. The DDSM and mini-MIAS dataset were used to access the mammogram images. The outcomes show that the FADHECAL technique had better results than the other selected enhancement methods, with AMBE = 6.502 ± 1.855, SSIM = 0.934 ± 0.034, MAE = 15.742 ± 1.217, PSNR = 26.843 ± 2.541, UIQI = 0.969 ± 0.021, and RMSE = 1.151 ± 0.147. The FADHECAL technique can be used to enhance mammogram images to detect breast cancer lesions more accurately with a reduced level of noise while preserving image detail.

Several approaches are presented in [59] to enhance mammographic contrast, including classical methods (linguistic hedges and fuzzy enhancement functions), advanced fuzzy sets (intuitive, Pythagorean, and Fermatean fuzzy sets), and genetic algorithm optimization. An advanced fuzzy set provides a more accurate assessment of the uncertainty of the membership function. For this reason, the intuitive method is the most efficient, but most of the other techniques are also effective, depending on the problem. Compared with conventional methods, linguistic methods can provide a more manageable way to spread the histogram, revealing more extreme values. It is possible to obtain a high-quality final image using ordered weighted averaging (OWA) operators combined with enhanced mammography images.

### 3.4. Assessing Breast Cancer Risk 

Due to the high incidence and mortality rates of breast cancer, the physical and mental health of breast cancer patients are adversely affected. There are numerous risk factors, such as age, family history, reproductive factors (e.g., early menarche, late menopause, first pregnancy at a late age, low parity), estrogen (e.g., endogenous or exogenous estrogen), and lifestyle (e.g., smoking, excessive alcohol consumption, dietary fat intake) [60]. Acknowledging and gaining a better understanding of breast cancer risks can promote early detection and prevention.

Numerous studies have extensively explored the use of AI in breast cancer risk prediction. For instance, in a systematic review of ML algorithms for breast cancer risk prediction reported from January 2000 to May 2018, Nindrea et al. found that the SVM algorithm had the highest accuracy compared to ANNs, decision trees, *k*-nearest neighbor, and naive Bayes algorithms [61]. Several other studies demonstrated that the combination of ANN and cytopathological diagnosis could be used to evaluate breast cancer risk by analyzing and learning mammography results, risk factors, and clinical findings, which can help doctors to make informed estimations of malignancy risk and improve the positive predictive value (PPV) of the decision to perform biopsy [62]. 

A number of studies [63,64,65,66,67] have employed large cross-sectional screening cohorts representing the general screening population to train DL models. These studies utilized normal mammographic images acquired at least one year before a breast cancer diagnosis or a negative follow-up (i.e., BIRADS 1 and 2). The design of these studies more closely reflects the task of assessing breast cancer risk, as they were aimed at identifying women at high risk before they develop cancer. It is important to use cases and controls of the same age in such a study and to report evaluation measures that are age-adjusted. Otherwise, risk prediction performance estimates can be inflated. The areas under the receiver operating characteristic curves (AUCs) for the models were in the range of 0.60 to 0.84, and they often outperformed state-of-the-art breast cancer risk models [59,60,63,65]. Ha et al. [67] showed that a full-field digital mammography (FFDM)-driven DL risk score was more predictive than the breast imaging and reporting data system (BI-RADS) breast density (odds ratios 4.4 vs. 1.7). FFDM-based DL risk scores outperformed automated breast density measurements according to Dembrower et al. [63]. Finally, Yala et al. found that a hybrid DL model involving both full-field mammograms and traditional risk factors recorded higher accuracy than the Tyrer–Cusick model, which represents the current clinical standard (AUC of 0.68 versus 0.62, respectively) [65]. These studies provide preliminary evidence that FFDM-based DL models offer promise for more accurate prediction of breast cancer risk than density-based models and existing epidemiology-based models. Given the above, the accuracy of AI techniques in the prediction of breast cancer risk is evident, which can undoubtedly help practitioners provide appropriate interventions to minimize the risk of breast cancer. Table 1 presents an overview of key AI papers in mammography.

## 4. Applications of AI in Ultrasound

Ultrasound is a diagnostic method with a high usage rate; this is due to its strengths of being free from radiation and easy to operate in addition to providing instantaneous results upon operation. Therefore, using ultrasound imaging to detect and diagnose breast cancer has become increasingly common. To address the need for quantification and standardization of ultrasound in order to avoid misdiagnoses (e.g., due to a lack of experience or subjective influence), the development of an AI system to detect and diagnose breast lesions in ultrasound images is proposed in [68]. Other studies [24,69,70] have demonstrated the use of AI systems to identify and segment RoIs, extract related features, and classify benign and malignant lesions in breast ultrasound images. Figure 2 shows ultrasound images of breast.

### 4.1. Identification and Segmentation of RoIs

Lesions must be identified and segmented from the background to determine an accurate representation for the diagnosis of breast lesions. At present, sonographers are responsible for manually segmenting breast ultrasound images. In addition to an experienced sonographer, this clinical process requires time and effort, and these images have poor contrast, unclear boundaries, and excessive shadowing. Therefore, an automatic segmentation method for breast ultrasound images is recommended. The segmentation process primarily involves detecting RoIs containing lesions and delineating the contours of the lesions. Hu et al. trained a combination of a phase-based active contour (PBAC) model and a dilated fully convolutional network (DFCN) and successfully achieved a high mean dynamic susceptibility contrast (DSC) of 88.97% in identifying and segmenting 170 breast ultrasound images, suggesting its effectiveness in guiding manual segmentation in medical analysis [71]. Kumar et al. demonstrated the performance of a multi-U-Net algorithm for segmenting masses in breast ultrasound images from 258 women, which surpassed the performance of the original U-Net algorithm, with a mean Dice coefficient of 0.82, a true positive value of 0.84, and a false positive value of 0.01 [72]. Feng et al. demonstrated that the performance of a Hausdorff-based fuzzy c-means (FCM) algorithm combined with an adaptive region selection scheme (involving the adaptive selection of the area around each pixel based on the mutual information between regions) for segmenting breast tumors in ultrasound images surpassed that of the Hausdorff-based and traditional FCM algorithms [73]. Using AI to automatically identify and segment breast lesions in ultrasound images can assist sonographers in accurately and efficiently detecting and diagnosing breast cancer.

Numerous researchers have delved into ultrasound-based breast cancer diagnosis, employing a range of methodologies. Early studies predominantly utilized traditional digital image processing techniques and machine learning approaches for detection. For instance, Drucker et al. [74] pioneered the use of radial gradient index filtering to identify initial points within regions, subsequently scrutinizing candidate areas against the background through the optimization of regional average radial gradient indices. Lesions were classified using Bayesian neural networks, yielding a sensitivity of 87%, with a false positive detection rate of 0.76.

Deep learning (DL), emerging as a prominent method in computer vision and pattern recognition, has garnered significant attention in medical research, including breast cancer detection [75]. Cao et al. [76] conducted a comprehensive comparison of five deep learning-based object detection networks, highlighting SSD’s superior performance in terms of precision and recall. In a study focusing on breast lesion detection, Yap et al. [77] employed Faster R-CNN as their deep learning network. To mitigate the impact of small sample datasets, they utilized transfer learning. Additionally, they introduced a three-channel fusion technique, merging original, sharpened, and contrast-enhanced images into a new three-channel image, enhancing detection accuracy.

Li Y et al. [78] developed BUSnet, a DL model, to analyze ultrasound images for the detection of breast tumors. First, a two-stage method was developed, which included a region proposal algorithm for unsupervised regions and a bounding box regression algorithm for supervised regions. A post-processing method was then proposed to further improve the detection accuracy. Using the proposed method, 487 benign samples and 210 malignant samples in a benchmark dataset were analyzed. The results showed that the proposed method proved to be effective and accurate.

A computerized analysis of breast ultrasound images for automatic breast tumor detection, classification, and volume estimation was developed in [79]. The Radiology Department at Thammasat University and the Queen Sirikit Center of Breast Cancer in Thailand provided breast ultrasound images. Among the 655 images, 445 were benign and 210 were malignant. The training and testing datasets were augmented through blur, flip vertical, flip horizontal, and noise transformations. The YOLO7 architecture, based on DL techniques, was then used for tumor detection, localization, and classification. A simple pixel-per-metric technique was used to estimate tumor volume. With a confidence score of 0.95, the model demonstrated excellent tumor detection performance, achieving 95.07% lesion classification accuracy, 94.97% sensitivity, 95.24% specificity, 97.42% PPV, and 90.91% NPV on the test sets.

Chorianopoulos et al. [80] applied three CNN models—MobileNet, VGG16, and AlexNet—to two datasets, one containing ultrasound images and the other containing histopathology images. On the ultrasound dataset, VGG16 achieved the highest accuracy of 96.82%. On the invasive ductal carcinoma dataset, MobileNet achieved the highest accuracy of 91.04%.

Byra et al. [81] proposed a deep-learning method for segmenting breast masses using ultrasound data. CNNs with selective kernels (SKs) were developed. The SKs adjusted the network’s receptive fields by combining dilated and conventional convolutions. Ultrasound images of 882 breast masses were used to create and evaluate the proposed method. Additionally, 893 ultrasound images obtained from three medical centers were tested. The SK-U-Net algorithm achieved a Dice score of 0.826 on 150 ultrasound images, outperforming the regular U-Net algorithm, which scored 0.778. A Dice score of 0.646 to 0.780 was achieved using the proposed method across three datasets.

An ultrasound image segmentation technique based on feature separation and complementation is presented in [82]. Top-to-bottom (T2B) and bottom-to-top (B2T) streams were used for feature separation, and each branch was much more effective at extracting the required feature information. Feature complementation was achieved by combining global semantic information with local detailed information at each stage. The result was a complementary boundary feature in the T2B stream, along with suppressed noise in the B2T stream. We evaluated these techniques using UDIAT, BUSIS, and LUSI, three publicly available datasets. Compared with other current methods for ultrasound image segmentation, the performance of our FSC-Net was at least 1.59%, 0.96%, and 3.74% better than other state-of-the-art methods.

### 4.2. Feature Extraction

Suspicious masses are typically identified and segmented according to the morphological and textural features in breast images, such as edge, echo type, hardness, orientation, rear features, shape, and location of calcification. Classification of suspicious masses according to the BI-RADS scale can quantify the degree to which cancer is suspected. Accurate identification of morphological features by sonographers is crucial for distinguishing between benign and malignant masses. Using AI systems for feature extraction from breast ultrasound images can assist in the diagnosis process, reducing the substantial demand on sonographers to deliver accurate diagnoses. Using FCM clustering, Hsu et al. found that combining morphological parameters (e.g., the standard deviation of the shortest distance), textural features (e.g., variance), and the Nakagami parameter allowed for high accuracy (89.4%), specificity (86.3%), and sensitivity (92.5%) when extracting physical features of breast ultrasound images. Conversely to using logistic regression and SVM classifiers, the maximum discrimination performance of the optimal feature collection did not depend on the classifier type, suggesting that the functional complementarity of combining different feature parameters can enhance the performance of breast cancer classification [83]. 

Zhang et al. combined feature learning and feature selection to form a two-layer DL architecture, which recorded an area under the receiver operating characteristic curve of 0.947 and had higher accuracy (93.4%), sensitivity (88.6%), and specificity (97.1%) when extracting and classifying shear wave elastography (SWE) features compared with the statistical features of quantified image intensity and texture [84]. The use of a CAD system (e.g., the Samsung RS80A S-Detect ultrasound system) has been reported to significantly improve the diagnostic performance of radiologists, regardless of their experience in analyzing the ultrasound features of breast masses. Using a CAD system can help to refine the description of breast lesions and enhance consistency among observers regarding the characteristics of breast masses, ultimately resulting in better decision-making [85]. 

Jabeen et al. [86] proposed a new framework for the classification of breast cancer based on ultrasound images by combining DL and best-selected features. The proposed framework includes five major steps: (1) data augmentation to enhance the size of the original dataset for learning CNNs; (2) modification of the output layer of the pre-trained DarkNet-53 model based on the augmented dataset classes; (3) training of the modified model by transfer learning and use of the global average pooling layer to extract features; (4) use of reformed differential evaluation, reformed grey wolf (RGW), and improved optimization algorithms to select the best features; and (5) use of a new probability-based serial approach and ML algorithms to fuse together and classify the best-selected features. In the experiment, the best accuracy was 99.1% based on augmented breast ultrasound images (BUSIs). The proposed framework performed better than other recent techniques.

Breast cancer data were successfully analyzed in [87] using LeNet, a classic CNN architecture. The system demonstrated high accuracy in early detection and diagnosis of breast cancer by extracting discriminative features and classifying malignant and benign tumors. As a result of addressing the “dying ReLU” problem and improving the discriminative power of the extracted features, LeNet with a corrected rectified linear unit (ReLU) demonstrated enhanced performance in breast cancer data analysis tasks, making breast cancer detection and diagnosis more accurate and reliable. LeNet’s training stability and performance can be improved through batch normalization. This method is useful in mitigating the effects of internal covariate shifts, which refers to changes in the distribution of network activation due to training. There will be reductions in the over-fitting problem and the running time with the use of this classifier. A comparison of the designed classifier to benchmark DL models showed that it had a higher recognition rate, with 89.91% of breast images recognized accurately.

### 4.3. Applications of AI in Thermography Images

DL has emerged as a powerful tool in the field of medical image analysis, including breast cancer classification from thermography images. Thermography, which captures the heat patterns emitted by the body, offers a non-invasive and radiation-free alternative to traditional imaging modalities. DL algorithms, such as CNNs, have demonstrated remarkable capability in extracting intricate patterns and features from thermal images. By training the model on diverse datasets, it learns to discern subtle temperature variations associated with malignant and benign breast tissue. The utilization of DL in thermography-based breast cancer classification holds promise for improving diagnostic accuracy and early detection, potentially enhancing the effectiveness of screening programs and contributing to more personalized and timely patient care [88]. 

Thermal images from the Database for Mastology Research with Infrared Images (DMR-IR) were employed in [89] to explore the effectiveness of VGG16, a pre-trained CNN architecture, in combination with attention mechanisms (AMs) for diagnosing breast cancer. The investigation focused on three variants of the model, each incorporating a distinct type of AM. The methodology revealed consistency in the performance of these models across all stages of the study. Notably, the test accuracy of the VGG16 model coupled with AMs on the breast thermal dataset demonstrated promising results, reaching 99.80%, 99.49%, and 99.32%. Compared to VGG16 without AMs, the test accuracy of VGG16 with AMs exhibited notable improvement of 0.62%, underlining the potential of using attention mechanisms to enhance diagnostic performance for classifying breast cancer in thermal images.

**Figure 3:**
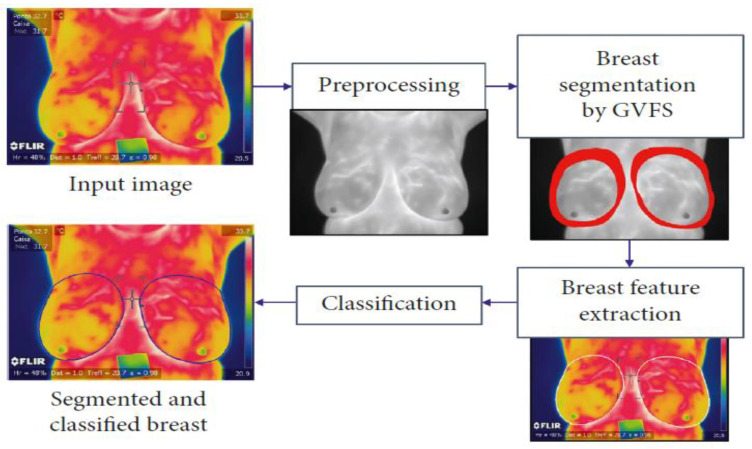
Proposed method for breast cancer segmentation from thermography images [88].

A two-stage model for breast cancer detection utilizing thermographic images is introduced in [90]. The first stage involves feature extraction from images using the VGG16 DL model. In the second stage, the dragonfly algorithm (DA), a metaheuristic algorithm, is used to select the optimal subset of features. To enhance the performance of the DA, a memory-based version incorporating the Grunwald–Letnikov (GL) method is proposed. The efficacy of the two-stage framework was assessed using the DMR-IR standard dataset. Impressively, the proposed model demonstrated the ability to efficiently filter out non-essential features, achieving a diagnostic accuracy of 100% on the standard dataset. Furthermore, it achieved this accuracy with 82% fewer features compared to the VGG16 model, highlighting the potential of the approach for improving both efficiency and accuracy in detecting breast cancer in thermographic images. Tello-Mijares et al. [88] focused on a segmentation method that combines the curvature function, k, and the gradient vector flow, while for classification they proposed a CNN using the segmented breast. The primary objective of the study was to compare the results of the CNN with other classification techniques. Each breast was characterized by its distinct shape, color, and texture and whether it was the left or right breast. These features were utilized to both train the models and evaluate the performance of the CNN against three other classification techniques: a tree random forest (TRF), a multilayer perceptron (MLP), and the Bayes network (BN). The findings revealed that CNN outperformed the TRF, MLP, and BN, demonstrating its superiority in breast characterization and classification based on shape, color, and texture features. Figure 3 shows the main stages of the proposed method for breast cancer segmentation from thermography images.

### 4.4. Benign and Malignant Classifications

**Figure 4:**
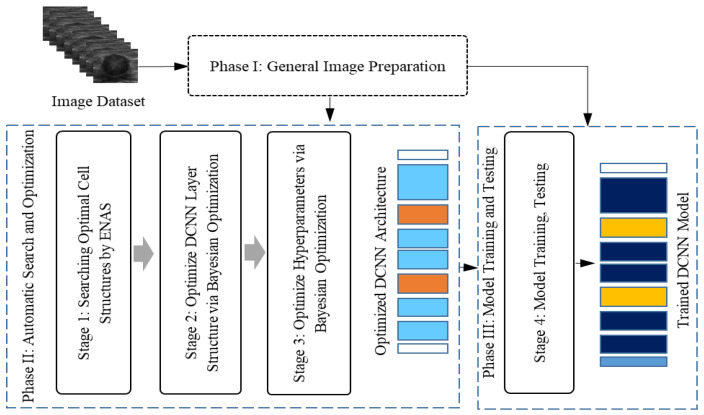
ENAS-B framework proposed in [96] for automatically designing a CNN model for breast cancer classification from ultrasound images.

Due to the high incidence and mortality of breast cancer among women globally, various measures have been developed to promote breast cancer screening for women of appropriate ages. The most significant aspect of breast cancer screening is detecting benign cases and malignant cases. Classification of breast lesions in ultrasound images is primarily based on the BI-RADS. In order to ensure consistency of the interpretations made by doctors with different experience levels, there have been growing efforts to develop AI systems for benign and malignant classification. Cirtisis et al. demonstrated that the use of a deep convolution neural network (dCNN) yielded comparable accuracy (93.1%; external: 95.3%) when classifying breast ultrasound images into a BI-RADS score of 2–3 and a BI-RADS score of 4–5 compared to the classification accuracy of radiologists (91.6 ± 5.4%; external: 94.1 ± 1.2%) [91]. Meanwhile, Becker et al. reported that a DL model trained on 445 cases had comparable accuracy when analyzing 637 breast ultrasound images (84 malignant lesions and 553 benign lesions) to that of a radiologist and better accuracy than that of a medical student (similarly trained with 445 cases) [92]. Recently, researchers started using automatic search methods to design CNN architectures from scratch for medical imaging, including breast cancer imaging. Ahmed et al. [93] used the efficient neural architecture search (ENAS) method to generate a model, which achieved 89.3% overall accuracy, outperforming other manually designed alternatives. Additionally, the ENAS-generated model had simplified complexity and greater efficiency. To investigate the generalization of the ENAS-based model [94], they evaluated the model on an external dataset. To address the challenge of generalization error, they investigated various techniques, such as reducing model complexity, employing data augmentation, and utilizing unbalanced training sets. The experimental findings indicate that the ENAS model trained on an unbalanced dataset with more benign images generalized well on the external dataset and two external datasets. Alzhoubi et al. [95] used a Bayesian optimizer as a search strategy to automatically search for CNN architecture for breast cancer classification. The result showed that the automatically generated CNN outperformed transfer learning CNN models on internal and external test sets. Ahmed et al. [96] proposed an automatic search environment for designing a CNN model for breast cancer classification from ultrasound images by combining the ENAS method with the Bayesian optimizer. Their method consists of two main steps: First, they used ENAS to generate optimal cells (normal and reduction), and second, they used Bayesian optimization to search for the number of cells per CNN architecture and trainable hyperparameters. The generated ENAS-B model outperformed the original ENAS and transfer-learning models in breast cancer classification. Figure 4 presents the proposed approach for automatically searching for CNN for breast cancer classification from ultrasound images.

The use of AI can significantly assist physicians in classifying and diagnosing benign and malignant cases in breast ultrasound images, particularly with regard to improving the diagnostic accuracy of inexperienced doctors. Table 2 presents key AI papers used ultrasound images for breast cancer.

## 5. Applications of AI in MRI Images

Magnetic resonance imaging (MRI) stands as a pivotal imaging modality in the comprehensive arsenal deployed for diagnosing breast cancer. With its ability to provide detailed anatomical images and delineate soft tissue structures with high precision, MRI plays a crucial role in detecting and characterizing breast lesions.

Slimani [97] introduced a groundbreaking method known as the 3D automatic level propagation approach (3D ALPA), a sophisticated technique devised to enhance the accuracy and efficiency of tumor volume reconstruction in breast MRI data. This innovative approach comprises two meticulously crafted steps. Firstly, the entire volume slated for processing undergoes segmentation, meticulously dissected slice by slice through a combination of global thresholding operations and morphological closure techniques. This step ensures the precise isolation of relevant structures within the volumetric data. Subsequently, in the second step, the segmented results from each slice are ingeniously fused together to meticulously reconstruct the original 3D volume housing the tumor, thus providing clinicians with a comprehensive representation for diagnosis and treatment planning. In a similar vein, Pandey et al. [98] pioneered a fully automatic and unsupervised approach by integrating the continuous max flow (CMF) method with sophisticated noise reduction algorithms and morphological operations. Their methodological innovation represents a significant stride towards automating the process of lesion detection and segmentation in breast MRI, streamlining clinical workflows and reducing dependency on manual intervention.

Furthermore, recent advancements in deep learning methodologies have catalyzed a paradigm shift in breast tumor targeting. The UNet architecture, a deep learning framework renowned for its efficacy in semantic segmentation tasks, has emerged as a frontrunner in automated breast lesion segmentation. Chen et al. [99] further pushed the boundaries by proposing an end-to-end network that harnesses both spatial and temporal resources, culminating in a fully automated approach to breast lesion segmentation. Their novel adaptation of the UNet architecture, coupled with the integration of ConvLSTM structures, demonstrates the potential for leveraging temporal information to enhance segmentation accuracy and robustness.

Moreover, Benjelloun et al. [100] and El Adoui et al. [101] pioneered innovative methodologies based on the UNet framework, tailoring their approaches to address the intricacies of segmentation within individual image slices. Their contributions not only underscore the versatility of deep learning frameworks but also highlight the nuanced challenges inherent in breast lesion segmentation tasks. In parallel, Lu et al. [102] and Santucci et al. [103] embraced the power of convolutional neural networks (CNNs), leveraging their inherent capability to learn complex patterns and extract meaningful features directly from input data. The utilization of CNNs, augmented with a softmax function in its outputs, enables seamless mapping of input images to final labels, obviating the need for laborious feature extraction steps. Additionally, their utilization of proprietary databases underscores the critical role of data accessibility and quality in training robust deep learning models for clinical applications.

## 6. Discussion

Despite tremendous advancements in the medical field over the past decade with the introduction of AI techniques, the integration and large-scale application of these techniques are still in the initial stages. CAD systems have several limitations regarding breast cancer screening, such as the existence of few large-scale public datasets, reliance on RoI annotation, high image-quality requirements, regional differences, and over-fitting and binary classification issues. Furthermore, AI techniques cannot handle multiple tasks concurrently, which can be challenging for the development of DL models for breast imaging. These issues are driving the development of breast imaging diagnostic discipline and reflect the broad prospects of intelligent medical imaging.

Apart from using AI techniques in conventional imaging methods, DL-based CAD systems have been under rapid development for digital breast tomosynthesis [104,105,106,107,108,109,110,111,112,113,114,115,116], ultrasound [107,108], and contrast-enhanced mammography. AI in breast imaging can be used to detect, classify, and predict breast diseases; classify specific breast diseases (e.g., fibroplasia); and even predict lymph node metastasis [109] and disease recurrence [110]. With technological advancements in the field of AI, the classification and diagnosis of breast diseases and the establishment of adjuvant treatment are expected to become more efficient and accurate, allowing for more effective early detection, diagnosis, and treatment for patients.

AI techniques, especially DL, have been increasingly used in medical imaging due to their promising potential and outstanding performance in analyzing medical images. AI techniques can deliver fast computing speeds with good repeatability and no fatigue, ensuring that doctors obtain highly accurate and objective information, thus helping to minimize their workload as well as misdiagnoses or missed diagnoses [111]. The use of CAD systems for breast cancer screening has been explored in numerous studies. These systems can reliably identify and segment breast lesions, extract and classify features, estimate breast disease and breast cancer risk, and evaluate treatment effects and prognoses regardless of the medical imaging method used (e.g., mammography, ultrasound, MRI, or other types of imaging) [112,113,114,115,116]. These CAD systems are highly promising and provide various advantages in terms of assisting doctors, optimizing resource allocation, and improving accuracy.

## 7. Conclusions

This paper has undertaken a comprehensive review of recent advancements in machine learning and deep learning techniques for the detection and classification of breast cancer. By exploring various methodologies employed across diverse medical image types, the study aimed to provide a thorough understanding of current approaches in breast cancer identification. Special emphasis was placed on traditional machine learning and deep learning methods, highlighting their significance in this domain.

Therefore, developing AI for breast cancer recognition faces numerous challenges, including limited and imbalanced datasets, the need for interpretable AI in medical decision-making, privacy concerns surrounding sensitive medical data, ethical considerations related to biases in data, the resource-intensive manual annotation of datasets, the constant evolution of technology requiring ongoing updates, and the necessity for regulatory compliance before deployment in clinical settings. Moreover, overcoming these challenges necessitates collaboration among researchers, healthcare professionals, regulators, and technology developers to create ethically sound and effective AI solutions for breast cancer detection and diagnosis.

## Figures and Tables

**Table 2:** Overview of key AI studies using ultrasound.

Ref.	Method	Application	Dataset Size	Accuracy
[71]	PBAC + DFCN	Segmentation	D1 570 cases, D2 128 cases	88.97%
[72]	Multi U-Net	Segmentation	433 cases	82%
[74]	RGI	Detection	757 cases	-
[75]	YoLov3	Detection	340 cases	76%
[76]	R-CNN, FastR-CNN, YoLov3, SSD	Detection	1043 cases	87.5%
[77]	Faster-RCNN with Inception-ResNet-v2	Detection	D-A 306 cases, D-B 163 cases	-
[78]	Region proposal algorithm and bounding box regression	Detection	697 cases	-
[79]	Yolo v7	Detection	655 cases	95%
[80]	CNN transfer learning	Classification	250 cases	96.82%
[81]	U-Net + SK	Segmentation	893 cases	82.6%
[82]	T2B and B2T	Segmentation	UDIAT 163 cases, BUSIS 184 cases	96%
[83]	Handcrafted ML	Classification	160 cases	89.4%
[84]	CNN	Classification	227 cases	93.4%
[85]	CNN transfer learning + (RGW)	Classification	200 cases	99.1%
[86]	CNN	Classification	343 cases	89.91%
[87]	dCNN	Classification	780 cases	93.1%
[93]	ENAS based CNN	Classification	524 cases	89.3%
[94]	Auto-Search CNN	Classification	2167 cases	85.8%
[95]	Auto-Search CNN and TL	Classification	3034 cases	83.33%
[76]	ENAS-Bayesian-CNN search	Classification	2624 cases	79.4%

## Data Availability

Data sharing not applicable.
